# Mitochondrial dynamics in macrophages: divide to conquer or unite to survive?

**DOI:** 10.1042/BST20220014

**Published:** 2023-02-23

**Authors:** Syeda Farhana Afroz, Karoline D. Raven, Grace M.E.P. Lawrence, Ronan Kapetanovic, Kate Schroder, Matthew J. Sweet

**Affiliations:** 1 Institute for Molecular Bioscience (IMB), The University of Queensland, Brisbane, QLD 4072, Australia; 2 IMB Centre for Inflammation and Disease Research, The University of Queensland, Brisbane, QLD 4072, Australia; 3 Australian Infectious Diseases Research Centre, The University of Queensland, Brisbane, QLD 4072, Australia; 4 Friedrich Miescher Institute for Biomedical Research, Basel, BS 4058, Switzerland

**Keywords:** inflammation, macrophages, mitochondrial dynamics, mitochondrial fission, mitochondrial fusion, neuroinflammation

## Abstract

Mitochondria have long been appreciated as the metabolic hub of cells. Emerging evidence also posits these organelles as hubs for innate immune signalling and activation, particularly in macrophages. Macrophages are front-line cellular defenders against endogenous and exogenous threats in mammals. These cells use an array of receptors and downstream signalling molecules to respond to a diverse range of stimuli, with mitochondrial biology implicated in many of these responses. Mitochondria have the capacity to both divide through mitochondrial fission and coalesce through mitochondrial fusion. Mitochondrial dynamics, the balance between fission and fusion, regulate many cellular functions, including innate immune pathways in macrophages. In these cells, mitochondrial fission has primarily been associated with pro-inflammatory responses and metabolic adaptation, so can be considered as a combative strategy utilised by immune cells. In contrast, mitochondrial fusion has a more protective role in limiting cell death under conditions of nutrient starvation. Hence, fusion can be viewed as a cellular survival strategy. Here we broadly review the role of mitochondria in macrophage functions, with a focus on how regulated mitochondrial dynamics control different functional responses in these cells.

## Introduction

Macrophages are innate immune cells with central roles in host defence in mammals. These cells constantly survey their surroundings, using pattern recognition receptors (PRRs) and other detection systems to sense and respond to indicators of danger, for example, infection or injury [1,2]. This results in the engagement of antimicrobial defence systems, coordination of inflammatory responses, priming of adaptive immunity, and initiation of repair processes. Macrophage-expressed PRRs recognise both exogenous pathogen-associated molecular patterns (PAMPs) such as components of microorganisms, as well as endogenous danger signals such as products released from dead or dying cells, tumour cells, and certain mitochondrial components that are collectively referred to as danger-associated molecular patterns (DAMPs). The innate immune system is equipped with diverse families of PRRs, including the toll-like receptors (TLRs), C-type lectin receptors, retinoic acid-inducible gene 1 (RIG-1)-like helicase receptors (RLRs), and nucleotide-binding oligomerization domain-like receptors (NLRs), with each family being comprised of several different receptors [3]. Despite this diversity, there is often overlap in the downstream biological responses that are generated upon sensing PAMPs and/or DAMPs. This may partly reflect the involvement of the mitochondrion, a key organelle integrating extracellular signals, cell metabolism, and biological outputs in macrophages.

The conception of mitochondria as a signalling organelle began with the discovery that the release of cytochrome c from mitochondria initiates a signalling cascade that leads to apoptotic cell death [4,5]. Since then, a vast literature has revealed that mitochondria have central roles in cell activation, cell survival, and many forms of cell death, with these organelles profoundly influencing numerous biological processes [6,7]. This has been extensively studied in immune responses, where mitochondria regulate both host defence [8] and sterile inflammation [9]. Mitochondria are dynamic organelles that can exist within a spectrum of morphological states within cells. This is governed by the cellular processes of mitochondrial fission and fusion, with the balance between fission and fusion often referred to as mitochondrial dynamics. Mitochondrial dynamics control many cellular pathways, including metabolism [10,11] and inflammatory responses [12–15]. In this review, we briefly describe the role of mitochondria in innate immunity, before focusing on how mitochondrial dynamics influence the metabolic status of macrophages, as well as the functional responses of these cells.

## Mitochondrial biology

Mitochondria are double membrane energy-generating organelles. The endosymbiont theory of mitochondrial origin proposes that a free-living α-proteobacterium was engulfed by an eukaryotic precursor cell ∼2 billion years ago, resulting in a mutually beneficial relationship [16]. During evolution, mitochondria lost most of the proteobacterial genomic materials and transferred many genes to the nuclear genome via endosymbiotic gene transfer [17]. Thus, most mitochondrial components are encoded by the nuclear genome. The small circular mitochondrial genome (mtDNA) mostly encodes translation machinery and components of respiratory chain complexes I, III, IV, and V for carrying out the key mitochondrial function of oxidative phosphorylation (OXPHOS), via the co-ordinated actions of the tricarboxylic acid (TCA) cycle and the electron transport chain (ETC). ETC complexes I, II, and III also generate mitochondrial reactive oxygen species (mROS) which contribute to various functions in innate immunity (see ahead).

## Functions for mitochondria in innate immunity

**Figure 1.**
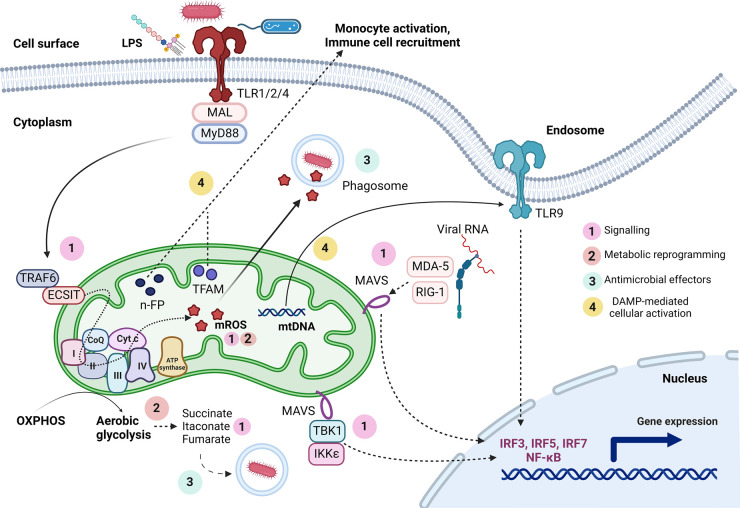
The multifaceted roles of mitochondria in innate immunity. Mitochondria have diverse functions in innate immune cells, including: (1) cell signalling, as exemplified by RLR-mediated engagement of MAVS for antiviral gene expression and TLR-inducible activation of ECSIT via TRAF6, as well as mROS and mitochondria-derived metabolites acting as signalling molecules; (2) metabolic reprogramming, as is apparent during TLR activation in which there is a metabolic shift from OXPHOS to glycolysis, as well as increased production of succinate, itaconate, fumarate, and mROS, all of which have inflammatory and/or antimicrobial roles; (3) generation of antimicrobial responses, with the antimicrobial effector molecule mROS and antibacterial metabolites all being produced downstream of TLR activation; and (4) DAMP-mediated cellular activation, in which mitochondrial DAMPs, such as TFAM, n-FP, and mtDNA, can all trigger innate immune activation. mtDNA, mitochondrial DNA; n-FP, N-formyl peptides; TFAM, mitochondrial transcription factor A. Created with BioRender.com.

Beyond their roles in energy generation, mitochondria control diverse cellular processes. In innate immune cells, mitochondria serve as signalling platforms for some PRR pathways, control PRR-inducible metabolic reprogramming, generate free radicals and metabolites that contribute to host defence and inflammation, and provide a reservoir of DAMPs for cellular activation upon disruption of homeostasis (Figure 1). Below we briefly describe examples of each of these.

Mitochondria are intimately connected to many innate immune signalling pathways. One of the most intensely studied examples of this involves the adaptor protein mitochondrial antiviral signalling protein (MAVS) that initiates antiviral responses upon RLR-mediated sensing of cytosolic viral RNA. MAVS is positioned at the mitochondrial outer membrane (OMM) where it forms complexes with the RLRs RIG-I [18] and MDA5 [19] upon activation by viral RNA. This interaction consequently triggers antiviral responses via the transcription factors interferon (IFN) regulatory factor (IRF) 3, IRF7, and nuclear factor-κB (NF-κB), leading to inducible expression of type I IFNs and other antiviral genes [20,21].

The burgeoning field of immunometabolism encompasses the role of mitochondria-regulated metabolism and metabolites in modulating the immune functions of cells, such as macrophages. Mitochondria-mediated metabolic changes alter macrophage functions, particularly their inflammatory and antimicrobial status. In response to lipopolysaccharide (LPS) and other inflammatory stimuli, cells rewire their metabolism from OXPHOS towards aerobic glycolysis, leading to a metabolic shift. For instance, LPS-inducible TLR4-activation redirects metabolic fluxes to generate acetyl-coenzyme A from glucose and increases ATP-citrate lyase activity, thus facilitating inducible histone acetylation in macrophages [22]. Moreover, Jha et al. [23] showed that the metabolites succinate and itaconate accumulate in activated macrophages due to a TLR-inducible break in the TCA cycle. Intriguingly, several studies have revealed that these metabolites have immunomodulatory and/or antimicrobial properties [24–28], though further studies are required to understand the *in vivo* relevance of some of these effects. One possible mechanism underlying metabolic reprogramming could be the translocation of TLR signalling molecules such as ECSIT [29] and STAT3 [30] to mitochondria in macrophages.

Under steady-state conditions, the mitochondrial ETC generates a small amount of ROS; however, this is amplified during cell stress and/or during metabolic adaptations. In macrophages, for example, LPS-inducible metabolic reprogramming leads to succinate accumulation that drives mROS production [25]. The increased mROS can activate pro-inflammatory signalling pathways [31,32], with this linked to many inflammatory conditions, for example, chronic obstructive pulmonary disease [33], chronic kidney disease [34], and type-1 diabetes-associated vascular inflammation [35]. Furthermore, TLR-inducible mROS also contributes to macrophage antibacterial responses [29,36]. Such studies have established mROS as an effector molecule of innate immunity.

Owing to their bacterial origin, mitochondria contain DAMPs, such as mtDNA, N-formyl peptides (n-FP), and mitochondrial transcription factor A (TFAM). Release of mitochondrial contents from damaged or necrotic cells can thus initiate sterile inflammation. For example, the concomitant release of n-FP and TFAM from necrotic cells activates monocytes [37] and promotes immune cell recruitment [38], while circulating mtDNA can trigger TLR9-mediated inflammatory responses [39,40] in cardiovascular-related conditions [41,42]. In this way, mitochondrial components can drive innate immune inflammatory responses.

## Mitochondrial dynamics: the interplay between fission and fusion

Mitochondria are dynamic organelles that exist in a continuum of states ranging from long filamentous to small spherical structures. The opposing processes of mitochondrial fission and fusion, referred to as mitochondrial dynamics, co-ordinate, and determine the overall mitochondrial morphology in a cell at any given time [43]. Mitochondrial dynamics play a vital role in mitochondrial quality control, cell division, and cellular stress responses. Underlying the importance of this process, the genetic deletion of essential regulators of mitochondrial dynamics results in embryonic lethality in mice [44,45]. For example, mice defective in genes required for mitochondrial fusion die in mid-gestation [44], while Wakabayashi et al. [45] demonstrated genetically that fission is essential for mouse embryonic and brain development, as well as mitochondrial morphogenesis, mitotic division, and cell death. In a healthy undisturbed cell, the balance in mitochondrial dynamics is generally skewed more towards a fused interconnected network of mitochondria, although fragmented spherical mitochondria are also normally present. When nutrients are limiting, the mitochondrial pool becomes hyperfused to enable functional cooperativity between mitochondria and cellular protection [46,47]. Conversely, excess nutrients and other stress signals lead to a hyperfragmented mitochondrial population, with fission exceeding fusion. This can have various functional consequences, including initiating apoptosis [48], aiding in metabolic adaptations [49], and regulating energy expenditure [50].

Cells use a specialised set of mechanical GTPases to control mitochondrial dynamics. One such GTPase, dynamin-related protein 1 (DRP1), encoded by *DNM1L*, is essential for mitochondrial fission [51,52]. DRP1 is a cytosolic protein that localises to mitochondria, forming an oligomeric complex upon activation. The act of fission occurs in two sequential steps. First, the endoplasmic reticulum (ER) and actin collaborate to mark a scission site where DRP1 assembles on the OMM. Next, DRP1 monomers form a large oligomer encircling this site, with the GTPase activity of DRP1 then facilitating membrane scission [53–56]. A recent study showed that the ER transmembrane protein CTRP1 directly interacts with DRP1 and facilitates its recruitment to mitochondria, suggesting a mechanism of ER–mitochondrial interaction during the initial stages of fission [57]. Several OMM-localised adaptor proteins have also been implicated in regulating DRP1-dependent fission. These include mitochondrial fission factor (MFF), mitochondrial dynamics of 51 kDa protein (MiD51), MiD49, and mitochondrial fission protein 1 (FIS1) [58–61]. DRP1 can bind to each of these adaptor proteins on the OMM, with the exact mechanisms by which they act being an intense area of current investigation.

MFF can directly bind to DRP1 to facilitate its recruitment, with the absence of MFF in HeLa cells skewing cells towards fusion [60,62]. There are contrasting studies on MiD49- and MiD51-mediated control of mitochondrial dynamics, with evidence that they promote both fission and fusion in different cell types [61,63,64]. Similarly, there may be context-dependent roles for FIS1 in mitochondrial fission. Zhang et al. [65] showed that FIS1 competitively binds to MiD51, suppressing its inhibitory effect on DRP1 to promote mitochondrial fission in a human lung-adenocarcinoma cell line. In contrast, Otera et al. [62] reported that FIS1 was dispensable for fission in HeLa cells. Kleele et al. [66] recently provided key insights into how different adaptor proteins regulate mitochondrial fission in different contexts to enable distinct functional outputs. Specifically, two distinct forms of DRP1-dependent fission were reported, one occurring at the periphery and another at the midzone of mitochondria. Peripheral fission occurs during mitochondrial stress and requires the establishment of FIS1-mediated lysosomal–mitochondrial contact sites. In contrast, midzone fission occurs during mitochondrial proliferation and requires MFF, along with ER-and actin-mediated pre-constriction of mitochondria. In this way, different DRP1 adaptor proteins can engage fission for distinct biological responses, namely quality control of mitochondria and cell division.

Both recruitment of DRP1 to mitochondria, along with its activation, are controlled by several post-translational modifications (PTMs). These include phosphorylation, S-nitrosylation, sumoylation, acetylation, and ubiquitination of specific residues. The contributions of specific PTMs on DRP1 to its activation and functional responses in different cell types are summarised in Table 1. This summary table highlights the diversity in DRP1 PTM sites, as well as in the enzymes involved in mediating these effects in different cell types. It is likely that different PTMs on DRP1 may influence its interactions with different adaptors for initiating or constraining fission, an area of investigation that is still evolving. For example, UV-stimulation of human lung-adenocarcinoma cells decreased phosphorylation of DRP1 at serine (S) 637, thus promoting a DRP1-MFF interaction and enhancing fission during apoptosis [65].

**Table 1.** PTM sites on DRP1, along with mechanisms involved (serine, S; alanine, A; threonine, T; cysteine, C; lysine, K; aspartic acid, D; glutamic acid, E; arginine, R)

Type of PTM	PTM site	Responsible enzyme	Effect on DRP1 activity	Specific DRP1 point mutations assessed	Cell type	References
Phosphorylation	S616	CDK1/cyclin	Activation	S to A	HeLa cells, human liver cells	[125,126]
		PKCδ	Activation	—	Mouse cardiomyocytes	[127]
		ERK2 (also known as MAPK1)	Activation	S to A	HEK-TtH cells	[128]
				S to A	Huntington's disease mouse striatal cells	[129]
		PINK1	Activation	S to A S to D	HEK293 cells	[130]
				S to A S to D	Mouse primary neurons	[131]
		CDK5	Activation	S to A S to E	Glioblastoma cells	[132,133]
			Inhibition	S to A S to D	Mouse primary neurons	[134]
		Ca^2+^/calmodulin-dependent kinase II (CaMKII)	Activation	S to A	Rat cardiomyocytes	[135]
	S412 S684	TBK1	Inhibition	S to A S to D	HEK293T cells	[136]
	S637	PKA	Inhibition	S to A S to D	Rat PC12 cells, African green monkey kidney fibroblast cells	[137]
		CaMKIa	Inhibition	S to A S to D	Rat primary neurons, HeLa cells	[138]
	T595	LRRK2	Activation	T to A T to D	HeLa cells, HEK293T cells	[139]
Dephosphorylation	S637	Calcineurin (also known as PP2B)	Activation	S to A S to D	Rat PC12 cells, African green monkey kidney fibroblast cells	[137]
		Neuron-specific PP2A/Bβ2 phosphatase	Activation	—	Mouse hippocampal neurons	[140]
S-nitrosylation	C644	Redox-mediated catalysis (donor is nitric oxide)	Activation	C to A	Mouse cerebrocortical neurons	[141]
	Not indicated	Protein disulphide isomerase	Facilitates DRP1 S616 phosphorylation and activation	—	Mouse hippocampal neurons	[142]
Sumoylation	Not indicated	SUMO E3 ligase, MAPL	Activation	—	HeLa cells	[143]
De-sumoylation	Not indicated	SENP5	Inhibition	—	COS-7 murine fibroblast like cells	[144]
	K557, K560, K569 or K571	SENP3	Activation	K557, 560, 569, and 571 to R	Mouse primary cortical neurons	[145]
			Enhanced DRP1-MFF binding	K557, 560, 569, and 571 to R	HEK293 cells	[146]
Ubiquitination	Not indicated	E3 ubiquitin ligase, MARCH	Activation	—	HeLa cells	[147]
			Inhibition	—	COS-7 murine fibroblast like cells, HeLa cells	[148,149]
Acetylation	K642	Not identified yet	Activation	K to R	Mouse cardiomyocytes	[150]

In comparison with fission, fusion requires more stringent regulation by multiple GTPases, both at the OMM and the IMM [67]. The IMM lipid cardiolipin interacts with the GTPase optic atrophy 1 (OPA1) to promote its GTPase activity [68,69], enabling it to initiate IMM fusion. In contrast, the GTPases mitofusin 1 (MFN1) and MFN2 drive OMM fusion [44,68,70]. In addition, two OMM proteins, FAM73a and FAM73b, facilitate fusion downstream of MFNs via the mitochondrial phospholipase D [71]. The fusion-promoting GTPases are also regulated via distinct PTMs. For example, MFN1 and OPA1 deacetylation by the lysine deacetylases HDAC6 [72] and sirtuin 3 [73], respectively, activate these GTPases to promote mitochondrial fusion. In contrast, MFN1 phosphorylation results in its ubiquitin-mediated proteasomal degradation, thus inhibiting fusion [74]. Given the diverse regulatory mechanisms that control each GTPase involved in fission and fusion, it is evident that complex mechanisms connect cell signalling to mitochondrial dynamics, with much yet to be understood about how mitochondrial dynamics are regulated.

## Regulated mitochondrial dynamics in macrophages

**Figure 2.**
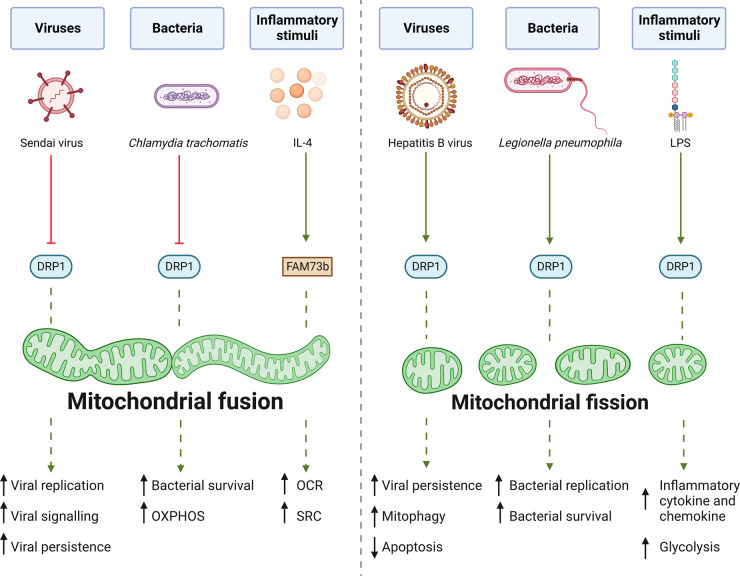
Modulation of mitochondrial dynamics by inflammatory stimuli and infectious agents. Pathogens and inflammatory stimuli can regulate mitochondrial dynamics, driving either mitochondrial fusion or fission depending on the pathogen/stimulus and cellular context. Specific examples of viruses, bacteria, and inflammatory stimuli that drive either mitochondrial fusion or fission are shown. These can affect mitochondrial dynamics through a variety of mechanisms, with modulation of DRP1 being common to many stimuli/pathogens (with the exception of IL-4, which skews towards fusion via the mitochondrial outer membrane protein FAM73b). Pathogen-driven manipulation of mitochondrial dynamics to either fusion or fission can favour pathogen persistence, replication, and/or survival, depending on the nature of the pathogen and cellular context. Red arrows indicate inhibition, green arrows indicate activation, dotted green arrows indicate positive effect on either fusion or fission. OCR, oxygen consumption rate; OXPHOS, oxidative phosphorylation; SRC, spare respiratory capacity. Created with BioRender.com.

Several innate immune stimuli and pathogens modulate and/or disrupt mitochondrial dynamics (Table 2), with this having many consequences for cellular functions (Figure 2). Given the range of stimuli that can affect fission and fusion, it seems likely that multiple PRRs and PRR signalling pathways may converge to modulate mitochondrial dynamics. The consequences of this modulation on macrophage metabolism, inflammatory outputs, phagocytosis, and the host–pathogen dynamic, are discussed below.

**Table 2.** Modulation of mitochondrial dynamics by innate immune stimuli

Stimuli	Cell type	Effect on fission or fusion	Functional consequences	Evidence	References
*Extracellular signals*					
LPS	Murine macrophages. Murine microglial cells	Fission **↑**	Inflammatory cytokines **↑**	*Drp1* silencing or treatment with Mdivi1	[10,12,81]
Succinate	Rat cardiomyocytes	Fission **↑**	Cell apoptosis, myocardial ischaemia injury	DRP1 recruitment to mitochondria and activation of MFF	[127]
IL-4	Murine macrophages	Fusion **↑**	OXPHOS**↑**	Mitochondrial morphology, *MFN1* and *MFN2* ↑	[81]
TNF	H9C2 cardiomyocytes	Fission **↑**	Cell death during sepsis **↑**	Inhibition of DRP1 by Rho-associated kinases inhibitor	[151]
Poly(I:C)	HEK293T cells	Fusion **↑**	Cell survival	TBK1 inhibits mitochondrial aggregation of DRP1	[136]
*Bacterial infections*					
*Shigella flexneri*	HeLa cells	Fission **↑**	Cell death **↑** Cell-to-cell spreading **↑**	*DRP1* silencing or treatment with Mdivi1	[152]
*Legionella pneumophila*	Human macrophages	Fission **↑**	Glycolysis **↑** Bacterial survival **↑**	DRP1 inhibition with Mdivi1	[112]
*Chlamydia trachomatis*	HUVECS, HeLa cells	Fusion **↑**	OXPHOS **↑** Bacterial survival **↑**	DRP1 levels **↓**	[153]
*Vibrio cholerae*	HEK cells, CHO cells, HeLa cells	Fission **↑**	Host inflammatory responses **↑**	Bacterial VopE interacts with Miro GTPases at mitochondria	[154]
*Listeria monocytogenes*	HeLa cells	Fission **↑**	ATP production **↓** Bacterial survival **↑**	Genetic silencing of *DRP1, MFN1* and *MFN2*	[111,155]
*Helicobacter pylori*	Human epithelial AZ-521 cells	Fission **↑**	Cell apoptosis **↑**	DRP1 inhibition with Mdivi1	[156]
*Viral Infections*					
Dengue virus	Human hepatoma 7 cells	Fusion **↑**	Viral replication **↑**	DRP1 expression **↓**	[157,158]
Sendai virus	HEK293T cells, HeLa cells	Fusion **↑**	Viral persistence, virus detection and signalling **↑**	*DRP1, FIS1*, *OPA1* and *MFN1* silencing	[159]
Venezuelan equine encephalitis virus	U87MG (human glioblastoma cell line)	Fission **↑**	Mitophagy, autophagy and cell death **↑**	Inhibition of fission with Mdivi1	[160]
Epstein–Barr virus	Gastric and breast cancer cells	Fission **↑**	Cell apoptosis and migration **↑**	DRP1 levels **↑**	[161]
SARS coronavirus	Pulmonary epithelial cells, HEK cells, THP-1 cells	Fusion **↑**	Innate immune signalling **↓** Viral persistence **↑**	DRP1 levels **↓**	[162]
Influenza A virus	HEK cells, murine macrophages	Fission **↑**	Antiviral response **↓**	Influenza A viral protein PB1-F2 localises to mitochondria	[163,164]
Hepatitis B virus	Human hepatoma 7 cells	Fission **↑**	Mitophagy **↑** Apoptosis **↓** Viral persistence **↑**	DRP1 S616 phosphorylation, MFN2 ubiquitination and degradation	[165]
Hepatitis C virus	Human hepatoma 7 cells	Fission **↑**	Viral persistence **↑** Apoptosis**↓**	DRP1 S616 phosphorylation and translocation to mitochondria	[166]

## The link between mitochondrial fission and metabolic reprogramming

Alterations in mitochondrial dynamics are interwoven with changes in the metabolic phenotype of a cell. When fusion is favoured over fission, cells generally occupy a catabolic state and generate ATP through OXPHOS [49]. In fibroblasts, fusion was shown to have a causative role in promoting OXPHOS, with this required for cell proliferation [75]. In contrast, a hyperfragmented mitochondrial pool portrays an anabolic state and a shift towards aerobic glycolysis [76]. In cancer cells, one of the rate-limiting enzymes of glycolysis, pyruvate kinase isoform M2, directly binds to MFN2. This interaction results in augmented mitochondrial fusion and a subsequent metabolic shift towards OXPHOS, in this case leading to suppression of cancer cell growth [77]. On the contrary, Nair et al. [10] showed that LPS induces mitochondrial fission and skews metabolism from OXPHOS to glycolysis in primary microglia. They demonstrated that pharmacological inhibition of mitochondrial fission with Mdivi1 [78] reversed this metabolic reprogramming and attenuated LPS-induced pro-inflammatory cytokine and chemokine production in these cells. Similarly, Zhang et al. [11] showed that genetic silencing of *DRP1* inhibited LPS-inducible glycolysis in airway smooth muscle cells, as well as cell proliferation. Thus, growing evidence connects TLR-inducible mitochondrial fission to metabolic reprogramming.

## Mitochondrial dynamics and macrophage inflammatory responses

As discussed above, mROS and mitochondrial metabolites regulate macrophage inflammatory responses. Given the intricate link between mitochondrial dynamics and metabolism, current research in this area is dissecting the role of mitochondrial dynamics in inflammation. Most studies in this area have primarily focused on neuroinflammation [13] and neurodegenerative diseases [79] (see ahead). However, several *in vitro* studies using the primary mouse or human macrophages have investigated specific molecular pathways and inflammatory outputs. For example, LPS triggered mitochondrial fission in both primary human and mouse macrophages, with genetic or pharmacological targeting of DRP1 in mouse macrophages and embryonic fibroblasts inhibiting the LPS-inducible production of a subset inflammatory mediators including IL-12p40, IL-6, and TNF [12]. Gao et al. [80] also established that LPS- or *Staphylococcus aureus*-mediated activation of DRP1 in mouse macrophages facilitated the production of the pro-inflammatory cytokine TNF. Furthermore, depletion of the fusion-promoting protein FAM73b skewed towards fission, impaired OXPHOS and promoted specific TLR-induced pro-inflammatory responses in murine macrophages and dendritic cells [81]. This resulted in increased *Il12a* expression, as well as decreased *Il10* and *Il23a* expression, enhancing macrophage-mediated anti-tumour immune responses [81]. Similarly, genetic silencing of *MFN2* in primary human macrophages enhanced TLR2-mediated pro-inflammatory outputs [82]. However, *MFN2*-silenced cells showed only a mild mitochondrial fragmentation, with this attributed to compensatory expression of *MFN1* in the absence of *MFN2*. In contrast, Tur et al. demonstrated that *Mfn2*-deficient mouse macrophages were defective in LPS-inducible production of pro-inflammatory cytokines and nitric oxide (NO). However, they did not ascribe this phenotype to defective mitochondrial fusion, rather reduced ROS production. Interestingly, MFN2 was also shown to be essential for inflammasome activation upon RNA virus infection in mouse macrophages [83], suggestive of a role for mitochondrial fusion in this PRR pathway. In line with this, skewing towards fusion by silencing *Drp1* in murine macrophages increased ERK signalling, leading to subsequent activation of the NLRP3 inflammasome pathway and IL-1β release [84]. These studies on MFN2 are suggestive of pro-inflammatory functions for fusion, contrasting with the general view that fission and fusion are linked to pro- and anti-inflammatory responses, respectively. However, it is also possible that MFN2 may have an additional mitochondrial fusion-independent function that may account for these phenotypes. Overall, a growing body of literature has demonstrated that TLR agonists and other inflammatory stimuli alter mitochondrial dynamics (Table 2), with consequent initiation of specific inflammatory responses in macrophages. It should be noted that much of the existing literature on TLR-regulated mitochondrial dynamics has focused on TLR4, however, with additional studies now being required to ascertain whether other TLRs influence this cellular process and downstream biological effects.

## Mitochondrial dynamics and neuroinflammation

As noted above, much of the literature on mitochondrial dynamics and inflammation has focused on neuroinflammation, particularly with respect to microglia. These tissue-resident macrophages of the central nervous system regulate neuronal survival [85], tissue-repair [86], and immunity [87]. However, during infection or injury, microglia may adopt a pro-inflammatory phenotype, releasing cytokines, ROS, and NO [88]. Sustained and chronic release of these inflammatory mediators in the central nervous system is neurotoxic, and may promote neuronal damage [89]. For example, activated microglia are associated with initiating pro-inflammatory signalling to promote neuronal damage in several neurodegenerative diseases, including Parkinson's disease (PD) [90–92] and Alzheimer's disease [93]. Mounting evidence implicates pro-inflammatory microglia in neuroinflammation and neurodegenerative pathology.

Exactly how microglia drive neuroinflammation remains elusive, but several lines of evidence support a role for an axis involving TLR4 and mitochondrial fission. Intraperitoneal injection of LPS initiated microglial activation, as well as dopaminergic neuron degeneration in mice [94,95]. This suggests that microglial TLR4-mediated pro-inflammatory pathways can drive neurodegeneration. Several studies also showed that LPS drives fission in microglia, with this linked to increased ROS, NO, and pro-inflammatory cytokines (IL-1β, IL-6, TNF) [14,96,97]. Furthermore, inhibition of DRP1 function dampened inducible LPS-induced mRNA expression of *Il1b*, *Il6*, and *Tnf*, as well as intracellular ROS production, in a mouse microglial cell line [98]. Metabolic reprogramming from oxidative phosphorylation to glycolysis is required for microglia to adopt a pro-inflammatory phenotype [99], and as noted above, mitochondrial fission was required for this metabolic switch in microglia [10]. These data thus suggest that TLR4-mediated mitochondrial fission may enhance pro-inflammatory phenotypes in microglia. Interestingly, increased mitochondrial fission has also been observed in pro-inflammatory astrocytes *in vitro* [15], suggesting a conserved role for mitochondrial fission across multiple cell types during neuroinflammation.

Another possible mechanism of mitochondrial fission perpetuating neuroinflammation is via enhanced microglial NLRP3 signalling. It is established that mitochondrial dysfunction primes and/or engages the NLRP3 inflammasome. For example, mROS and mtDNA trigger assembly and activation of the cytosolic NLRP3 inflammasome, as well as pro-inflammatory responses via IL-1β release and cell death [100–102]. In mouse macrophages, skewing towards fusion suppressed the release of the inflammasome-dependent cytokine IL-1β [12]. Furthermore, antagonising mitochondrial fission in PD models reduced brain tissue expression of NLRP3 and NLRP3 signalling components, which were otherwise elevated in the brain tissue of rats with a PD-like phenotype [103]. Similarly, intraperitoneal administration of the fission-inhibiting compound Mdivi1 in an acute kidney injury model in mice significantly down-regulated the expression of NLRP3 and inflammasome-related proteins in kidney tissue [104]. This suggests that mitochondrial fission may contribute to the priming of inflammasome responses during neuroinflammation, as well as other inflammatory conditions. Moreover, the administration of mitochondrial fission inhibitors *in vivo* was neuroprotective in several animal models of neurodegenerative disease. For example, intraperitoneal injection of Mdivi1 protected against dopaminergic neuron damage in a rat model of PD [105]. Similarly, another mitochondrial fission inhibitor, P110 [106], prevented the loss of dopaminergic neurons and improved motor ability in a PD mouse model [107]. The specific mechanisms involved are not well understood, but collectively these data suggest that mitochondrial fission may contribute to neuroinflammation and progressive neurodegenerative disease.

## Mitochondrial dynamics and phagocytosis

A few studies have documented key roles for mitochondrial dynamics in macrophage phagocytic responses [108–110]. Wang et al. [108] demonstrated that initial apoptotic cell uptake triggers DRP1-dependent fission in murine macrophages, with this facilitating continued clearance of the apoptotic cells. The importance of fission in this efferocytosis response was validated *in vivo* using myeloid-specific *Drp1*-knockout mice. Consistent with these findings, tumour cells resist phagocytosis by human macrophages by inhibiting mitochondrial fission in these cells, and this pathway can be targeted for effective antibody therapy against several malignancies [109]. In contrast with the pro-phagocytic activity of fission, the fusion-mediating protein MFN2 was also required for phagocytosis, as demonstrated using myeloid-specific *Mfn2-*knockout mice [110].

## Mitochondrial dynamics and host defence

As evident in Table 2, a wide array of pathogens can modulate mitochondrial dynamics, with the functional consequences of this being either detrimental or beneficial for the pathogen. This may reflect different roles for mitochondrial dynamics in different cell types, different kinetics, and/or the specific pathogen being studied. For example, *Listeria monocytogenes* skewed mitochondrial dynamics towards fission in HeLa cells transiently, with mitochondria shifting back towards a more fused state over time [111]. Depleting *MFN1* and *MFN2* in these cells prolonged fission and impaired *Listeria* survival, while depleting *DRP1* skewed towards fusion and favoured bacterial survival. Hence, it was postulated that the transient nature of mitochondrial fission in these cells may reflect pathogen subversion to support intracellular survival. In contrast with this study in HeLa cells, myeloid-specific *Mfn2*-knockout mice have fission-skewed macrophages and were more vulnerable to septic shock, as well as *L. monocytogenes* and *Mycobacterium tuberculosis* infection [110]. Similarly, *Legionella pneumophila* triggered mitochondrial fission and a shift towards aerobic glycolysis in human macrophages [112], with pharmacological targeting of DRP1 decreasing intracellular survival of this bacterial pathogen in these cells [78]. Such studies suggest that regulated mitochondrial dynamics may influence the host–pathogen dynamic and antimicrobial defence; however, there are major knowledge gaps regarding the underlying mechanistic details of this pathway and how it applies to different pathogens.

## Conclusions and future directions

Although various inflammatory stimuli can modulate mitochondrial dynamics, a detailed molecular understanding of how different PRR signalling pathways exert these effects in macrophages is yet to emerge. Based on the variety of regulated PTMs on DRP1 alone, it can be speculated that altered mitochondrial dynamics is rather a universal response to many stimuli; however, the precise mechanisms involved may depend on the specific PAMP-PRR signalling pathway and/or cell type. Deconvoluting these mechanisms will be an interesting area of future research. Furthermore, different mechanisms of DRP1 activation, for example through distinct PTMs, may alter mitochondrial dynamics in different ways to elicit distinct functional outcomes. This may also be achieved through regulated or cell type-specific expression of different *DRP1* transcriptional variants, of which there are many [113]. This gene regulation-mediated mechanism could also enable isoform-specific PTMs and/or functions of DRP1 [114], including differential interactions with OMM adaptor proteins such as MFF [115]. Of note, DRP1 can also shape and fragment other organelles, such as the ER and peroxisomes [116,117]. Thus, careful consideration should be taken before attributing specific biological effects to mitochondrial dynamics, based on DRP1 manipulation alone.

Another interesting research direction for the future involves the potential control of macrophage functions by intercellular transfer of mitochondria [118]. Tunnelling nanotubes for intercellular mitochondrial transfer have been studied in different contexts, such as between cancer and immune cells [119], as well as between mesenchymal stem cells and macrophages during acute respiratory distress syndrome [120]. Brestoff et al. [121] also reported immunometabolic cross-talk between adipocytes and macrophages to regulate metabolic homeostasis in obesity. A subsequent study showed that macrophages transfer mitochondria from white adipose tissue to distant organs, such as the heart, via the circulation to facilitate metabolic adaptation during nutrient stress [122]. An intriguing question in this regard is whether mitochondrial dynamics are affected during such intercellular mitochondrial transfer, both in the recipient and donor cells. Whether there is interplay between mitochondrial dynamics and mitochondrial nanotunnels [123] and/or inter-mitochondrial junctions [124] to regulate organelle behaviour and cell–cell communication will also be interesting areas of future investigation. Finally, the continued assessment of myeloid-specific knockouts of *Drp1*, *Mfn1*, *Mfn2*, and/or other genes controlling mitochondrial dynamics in different animal models of inflammatory and infectious diseases will be informative for understanding the *in vivo* functions of mitochondrial dynamics in macrophages in health and disease.

## Perspectives

- Mitochondria have multifaceted roles in innate immunity. Many inflammatory stimuli and pathogens regulate mitochondrial dynamics in macrophages.
- Regulated mitochondrial dynamics control metabolic and inflammatory responses in macrophages. In myeloid cells, mitochondrial fission drives inducible glycolysis, production of specific inflammatory mediators, neuroinflammation, and phagocytosis.
- Future investigations into mitochondrial dynamics in macrophages should focus on defining the precise molecular mechanisms by which innate immune stimuli modulate mitochondrial dynamics, the downstream mechanisms that link regulated mitochondrial dynamics to biological effects, and the contributions of mitochondrial fission and fusion to homeostatic and disease processes *in vivo*.

## Open Access

Open access for this article was enabled by the participation of the University of Queensland in an all-inclusive *Read & Publish* agreement with Portland Press and the Biochemical Society under a transformative agreement with CAUL.

## Abbreviations

DAMP: danger-associated molecular pattern
DRP1: dynamin-related protein 1
ECSIT: evolutionarily conserved signalling intermediate in Toll pathways
ETC: electron transport chain
FIS1: mitochondrial fission protein 1
IKK: inhibitor of nuclear factor-κB (IκB) kinase
IMM: inner mitochondrial membrane
LPS: lipopolysaccharide
MAL: MyD88-adapter-like
MAVS: mitochondrial antiviral signalling protein
MDA-5: melanoma differentiation-associated gene 5
MFF: mitochondrial fission factor
MFN: mitofusin
NLRP3: NOD-, LRR- and pyrin domain-containing protein 3
OMM: outer mitochondrial membrane
OXPHOS: oxidative phosphorylation
PAMP: pathogen-associated molecular pattern
PRR: pattern recognition receptor
PTM: post-translational modification
RLR: retinoic acid-inducible gene 1 (RIG-1)-like helicase receptor
ROS: reactive oxygen species
TBK1: TANK-binding kinase 1
TCA: tricarboxylic acid
TFAM: mitochondrial transcription factor A
TLR: Toll-like receptor
TRAF6: tumour necrosis factor receptor-associated factor 6
